# Substrate-Mediated Raw Material Grade Affects Sensory Quality, Chemical Composition, and Fungal Community of Fu Brick Tea

**DOI:** 10.3390/foods15010100

**Published:** 2025-12-29

**Authors:** Liangliang Zhao, Wenwen Fang, Xingchang Ou, Tian Huang, He Xie, Yang Liu, Zhonghua Liu, Silei Bai, Jianan Huang

**Affiliations:** 1 Key Laboratory of Tea Science of Ministry of Education, Hunan Agricultural University, Changsha 410128, China; zlliang1228@163.com (L.Z.); fwenn@stu.hunau.edu.cn (W.F.); xco118@sina.com (X.O.); xhhhhh2022@126.com (H.X.); lliuyang0630@126.com (Y.L.); zhonghua-liu-ms@hunau.edu.cn (Z.L.); 2 Yiyang Tea Factory Co., Ltd., Yiyang 413000, China; xiangyi58@163.com; 3 Yuelushan Laboratory, Changsha 410128, China; 4 State Key Laboratory of Tea Plant Germplasm Innovation and Resource Utilization, Hunan Agricultural University, Changsha 410128, China; 5 Key Laboratory for Evaluation and Utilization of Gene Resources of Horticultural Crops, Ministry of Agriculture and Rural Affairs of China, Hunan Agricultural University, Changsha 410128, China; 6 National Research Center of Engineering and Technology for Utilization of Botanical Functional Ingredients, Changsha 410128, China

**Keywords:** Fu brick tea, *Aspergillus cristatus*, microbial community, substrate-mediated fermentation, polyphenols, sensory quality

## Abstract

Fu brick tea (FBT) develops its characteristic qualities through fermentation, yet how variation in the chemical composition of raw dark tea (RDT) is associated with microbial succession and final tea quality remains unclear. In this study, three grades of RDT (premium-grade (1M), first-grade (2M), and second-grade (3M)) were processed into FBT under identical conditions to examine the relationship between initial composition, microbial community structure, and sensory attributes. Results revealed that high-grade RDTs (1M) contained higher levels of water extracts (WE, 36.35 ± 0.14 (%), *p* < 0.05), total polyphenols (TP, 14.93 ± 0.19 (%), *p* < 0.05), and free amino acids (FAA, 2.90 ± 0.03 (%), *p* < 0.05), promoting *Aspergillus* (96.06% in C1M, compared with 66.43% in C2M and 55.01% in C3M) dominance and resulting in brighter liquor with enhanced body and smoothness. Correlation analyses demonstrated a coherent sequence from substrate composition to microbial assembly and then to quality-related chemistry. WE, TP, and FAA were positively correlated with *Aspergillus* abundance and body and smoothness (*p* < 0.05), whereas soluble sugars correlated with *Rhodotorula* and sweetness (*p* < 0.05). These findings support a substrate-mediated association framework in which the chemical composition of RDT is closely aligned with microbial community structure and sensory differentiation during FBT fermentation, providing a scientific basis for raw material grading and fermentation management in dark tea production.

## 1. Introduction

Fu brick tea (FBT), a representative post-fermented dark tea in China, is widely recognized for its characteristic golden-flower appearance, mellow and thick mouthfeel, and unique fungal–floral aroma. Beyond its sensory appeal, FBT has been associated with multiple health-promoting benefits, including antioxidant, hypoglycemic, and lipid-lowering properties [1,2]. These distinctive features are largely attributed to its characteristic post-fermentation stage known as “flowering,” during which *Aspergillus cristatus* (syn. *Eurotium cristatum*) colonizes the brick and forms macroscopically visible “golden flowers”. The flowering stage represents a defining step that distinguishes FBT from other dark teas such as Pu-erh or Qingzhuan teas and has been recognized as a key determinant of its chemical and organoleptic profile [3,4,5].

During the flowering stage, complex microbial communities, dominated by *A. cristatus*, mediate a wide range of biochemical transformations that substantially reshape the chemical composition of the tea substrate [6,7,8]. Enzymes produced by *A. cristatus*, including oxidases, hydrolases, proteases, and glycosidases, convert the polyphenolic, proteinaceous, and carbohydrate matrices of raw dark tea (RDT) into smaller, more volatile or soluble molecules, thereby contributing to the characteristic mellow taste and fungal aroma [9,10,11,12]. Polyphenol oxidation and degradation, together with reactions involving amino acids and reducing sugars, jointly drive the formation of liquor color, mouthfeel, and aroma complexity [13,14,15]. Consequently, understanding the interplay between microbial activity and chemical reconfiguration has been a longstanding focus of research on FBT and related dark teas [10,16,17,18].

Despite substantial progress in characterizing the microbial ecology of FBT, current studies have predominantly focused on associating with final quality attributes, whereas the upstream influence of raw material matrix on microbial succession remains relatively under-explored. However, the upstream influence of the raw material matrix on microbial succession has not been systematically addressed. Several investigations have shown that variations of raw materials in tea cultivar, tenderness, or regional origin can affect microbial communities and fermentation trajectories in post-fermented teas [19,20,21,22]. However, these effects are easily confounded by differences in downstream processing conditions, such as fermentation humidity, temperature, or inoculation strategy, making it difficult to isolate the specific contribution of raw material composition to microbial community configuration [23,24]. Recent studies on Fu brick tea have begun to address interactions between substrate composition, microbial succession, and sensory quality. Integrated metabolomic and microbiome analyses indicate that variations in polyphenols, amino acids, and soluble sugars can modulate *A. cristatus* dominance and influence the formation of key flavor compounds [25,26]. Nevertheless, how specific compositional features of raw materials structure fungal community assembly during the flowering stage of FBT remains insufficiently resolved. Although studies in Pu-erh and Qingzhuan teas have ever demonstrated that the contents of total polyphenols, amino acids, and carbohydrates affect microbial growth and enzyme secretion [27,28], direct mechanistic evidence linking raw material chemistry to fungal succession in FBT is still lacking.

In the present study, the influence of raw material grade on the sensory characteristics, quality-related chemical composition, and fungal community structure of FBT produced under standardized conditions were investigated. Using three grades of RDT (1M–3M) as inputs, the effects of differences in water extracts (WEs), total polyphenols (TPs), flavonoids (FLAs), free amino acids (FAAs), and soluble sugars (SSs) on microbial configuration and sensory outcomes during flowering were evaluated. The fungal community was characterized by using ITS amplicon sequencing and culture enumeration of *A. cristatus*, and correlation analyses were performed to integrate chemical composition with sensory attributes. By elucidating substrate-mediated linkages between raw material grade, microbial assembly, and quality formation, this study provides mechanistic insight into how raw material chemistry governs fermentation outcomes and sensory differentiation in FBT, offering a scientific basis for raw-material selection and targeted fermentation control in dark tea production.

## 2. Materials and Methods

### 2.1. Materials and Reagents

Six dark tea samples were provided by the Yiyang Tea Factory Co., Ltd. (Yigang, China). The tea leaves were stored in a well-ventilated, light-proof, and odor-free environment. To ensure consistency in the processing, dark tea raw materials of different grades were standardly processed through steaming, pile fermentation, juice addition, pressing, flowering, and drying to produce Fu brick tea of appropriate density. Based on quality and fresh leaf tenderness, RDTs were classified into three grades: high-grade (a bud with two leaves, 1M), medium-grade (a bud with three leaves, 2M), and low-grade (a bud with four leaves, 3M). For each grade, three samples were taken, each weighing 500 g, and the resulting FBT was numbered C1M, C2M, and C3M, respectively. Three replicates were performed.

### 2.2. Sensory Evaluation

Six trained group members (three men and three women) from Hunan Agricultural University were selected to conduct sensory evaluations of tea samples. Prior to the formal experiments, each assessor underwent at least 90 h of specialized training to ensure they could match, rank taste, and accurately describe the sensory characteristics of tea. The sensory experiment was conducted in the tea evaluation room established at Hunan Agricultural University following the GB/T 18797-2018 [5,29]. A total of 3 g of tea sample was placed in a special cylindrical evaluation cup, added with 150 mL boiling water, covered, soaked for 5 min (raw dark tea) or 8 min (Fu brick tea), and strained into the evaluation bowl for evaluation. All the dark tea samples were evaluated in a blind manner and repeated in triplicates. During the evaluation, the assessor was required to evaluate the appearance, soup color, aroma, taste, and leaf base of each sample and to provide the key terms to describe quality characteristics collectively. In addition, quantitative descriptive analysis (QDA) would be used for sensory analysis in the taste reconstitution experiments. QDA will establish 6 sensory tastes, which are “Richness”, “smoothness”, “umami”, “sweetness”, “bitterness”, and “astringency”. The intensities of 6 sensory attributes in the tea soup were evaluated and described from 0 to 5, in which “0” represented as not perceivable, “3” as moderate, and “5” as strong. The evaluation environment requirements were that it was clean and odorless, with a temperature of 20–25 °C. The average value of the evaluation results of the six evaluators was considered the final result of the samples.

### 2.3. The Determination of Chemical Components

A total of 3 g of the dried RDTs and FBTs was extracted with 450 mL of distilled water bath for 45 min and then filtered.

The water extract (WE) content was determined according to GB/T 8305-2013 Tea—Determination of water extract content [6,30]. We accurately measured 50 mL of the tea infusion, filtered it through filter paper, and transferred it to an evaporating dish. The contents were evaporated to dryness over a boiling water bath. The residue was transferred to an oven at 120 °C and dried for 2 h. The residue was removed and placed in a desiccator to cool to room temperature before weighing.

Total polyphenol (TP) content was measured using the Folin–Ciocalteu method as specified in GB/T 8313-2008—Determination of total polyphenols and catechins content in tea [6,31,32]. Absorbance at 765 nm was measured (UV-2600, SHIMADZU, Kyoto, Japan). The content of polyphenols was determined from the calibration curve using gallic acid (GAE) as the reference standard (0–50 μg/mL of gallic acid).

Free amino acid (FAA) content was quantified following GB/T 8314-2013 Tea—Determination of free amino acids content [6,31,33]. The samples were shaken, heated at 100 °C for 15 min, cooled in ice water, and the absorbance at 570 nm was determined (UV-2600i, SHIMADZU, Kyoto, Japan). The results were expressed as mg glutamate (Glu) per mL of sample with respect to the standard curve including the dilution factor. The standard curve was first prepared using glutamate.

Soluble sugar (SS) content was analyzed by the anthrone–sulfuric acid colorimetric method [6]. We mixed 1 mL of each sample with 8 mL of 0.6% anthrone–sulfuric acid solution, shaken gently during addition. We used distilled water as a blank. The mixtures were incubated in boiling water for 3 min and cooled at room temperature (20 to 25 °C). The absorbance value was measured at 620 nm (UV-2600i, SHIMADZU, Kyoto, Japan). Glucose (25–250 μg/mL) in water was used for the calibration curve.

Flavonoid (FLA) content was determined via aluminum chloride colorimetry [6,30]. Different concentrations of flavonoids were used in the plotting of the standard calibration curve. Absorbance at 420 nm was measured (UV-2600, SHIMADZU, Kyoto, Japan). All assays were performed in triplicate.

### 2.4. Microbial DNA Extraction and Internal Transcribed Spacer (ITS) Sequencing

We suspended 15 g of tea samples in 150 mL sterile 0.1 M potassium phosphate (PBS) buffer (1:10 ratio, pH = 7.0), which was oscillated for at 28 °C for 30 min, filtered through three layers of coarse sterile gauze, and centrifuged at 13,400× *g* for 10 min at 4 °C. The precipitate was resuspended in 5 mL sterile PBS buffer and centrifuged again. Finally, the precipitate pellets were flash-frozen in liquid nitrogen and stored at −20 °C [34]. The microbial precipitate was used to extract DNA using the SPIN easy DNA Pro Kit for Soil (MPbio, Shanghai, China).

The extracted DNA samples were submitted to Guangzhou Genedenovo Bio-Technology Co., Ltd. (Guangzhou, China). for genome sequencing. DNA quality was assessed using agarose gel electrophoresis and other techniques. The full-length internal transcribed spacer (ITS) of fungi in tea was performed using the ITS3_KYO2 (5′-GATGAAGAACGYAGYRAA-3′) and ITS4 (5′-TCCTCCGCTTATTGATATGC-3′) universal primers. Subsequently, the NovaSeq 6000 system (Illumina, California, USA) was used for high-throughput sequencing of full-length ITS amplicons. All samples were tested for microbiota in three replicates. After passing quality control, sequencing libraries were constructed and subjected to quality inspection.

### 2.5. The Determination of Aspergillus Cristatus

The number of *Aspergillus cristatus* colonies in the FBT samples was counted according to the method specified in GB/T4789.15-2016 [35]. Fu brick tea weighing 25 g was added to a conical flask containing 225 mL of sterile potassium phosphate-buffered solution (containing glass beads). After shaking in a shaking incubator, we performed a serial dilution. The potato dextrose agar (PDA) medium (Huankai Microbiology, Guangzhou, China) was poured onto plates, and the plates were incubated upside down in a constant-temperature incubator at (28 ± 1) °C. The colonies were counted on the fifth day. The enumeration of microorganisms was performed in triplicate (by counting plates with 30–300 colonies) and the viable cell counts were expressed as CFU/g of the samples.

### 2.6. Statistical Analysis

All the assays were carried out with at least three biological replicates. The results are reported as the mean ± standard error of the mean (SEM). The normality and homogeneity of variances were tested using the Shapiro–Wilk test. SPSS Statistics 18.0 software (SPSS Inc., Chicago, IL, USA) was used to calculate significant differences based on a one-way analysis of variance (ANOVA), and Tukey’s HSD test was subsequently performed when significance effects were detected. The stacked bar charts were created using GraphPad Prism 8.0.2. Microbiota data were analyzed based on Operational Taxonomic Units (OTUs). Principal Co-ordinate Analysis (PCoA) and hierarchical clustering analysis (HCA) were performed using the Omicsmart online platform (www.omicsmart.com). The linear discriminant effect analysis (LEfSe) tool was used to analyze key differential fungus. First, Kruskal–Wallis tests (*p* < 0.05) were applied to detect taxa with significant OTU abundance differences across groups. Subsequently, linear discriminant analysis (LDA) was performed to estimate the effect size of each discriminative taxon (LDA score > 3.5). We screened for differentially expressed fungi with variable importance for the projection (VIP) > 1 and visualized results in a volcano plot, creating a Network Correlation Heatmap using Spearman correlation coefficient with the Mantel test using Omicshare online tools (www.omicshare.com).

## 3. Results

### 3.1. Sensory Evaluation Results of Different Grades of Raw Materials for Fu Brick Tea

Following GB/T 23776-2018 (panel training and evaluation procedures described in Methods), a trained panel evaluated raw dark teas (RDT;1M–3M) and the corresponding Fu brick tea (FBT; C1M–C3M). Results indicated consistent, grade-linked differentiation across appearance, liquor color, aroma, taste, and leaf base (Table 1).

**Figure 1:**
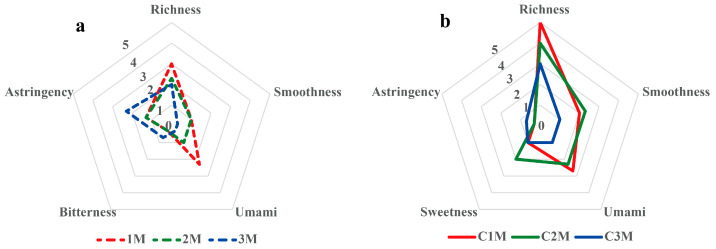
Radar plot of taste descriptors for (**a**) raw materials (RDT; 1M–3M) and (**b**) finished Fu brick teas (FBT; C1M–C3M). Panel means are shown for richness body, smoothness, umami, sweetness, bitterness and astringency on a 0–5 scale (GB/T 23776-2018); larger areas indicate more favorable profiles except for astringency (reverse-interpreted).

Sample 1M, representing the highest grade, exhibited tightly rolled, delicate leaves and produced a bright yellow-orange liquor with a pure, typical dark tea aroma and a mellow, full-bodied taste. Sample 2M yielded an orange-yellow liquor and a relatively pure aroma, with a modest reduction in perceived body compared with 1M. Sample 3M showed reduced aroma purity and noticeable astringency alongside only moderate mellowness, consistent with lower raw material quality within the tested set. The radar plot for RDT (Figure 1a) summarizes these differences: 1M shows higher scores for liquor brightness, aroma purity, body, and smoothness, whereas 3M shows lower scores in these attributes and higher astringency.

After standardized compression and post-fermentation (“flowering”), all finished products displayed typical morphology, flat brick surfaces with abundant golden “flowers” of *A. cristatus*, distinct edges, and moderate density, yet sensory differentiation persisted. C1M obtained the highest overall score, characterized by an orange-red, bright liquor; a pure and persistent fungus aroma; and a heavy, mellow, brisk, and smooth taste. The leaf base of C1M was uniform, soft, and tender, indicating a high degree of harmony. C2M ranked second, with a pure aroma and a thick, fresh, and smooth taste, although the fungus note was weaker than in C1M. C3M ranked lowest, presenting minor off-notes and higher perceived astringency that compromised harmony. The radar plot for FBT (Figure 1b) reflects this hierarchy (C1M > C2M > C3M) through systematically higher scores in body and smoothness and lower scores in astringency for C1M.

The sensory results showed clear grade-related trends for appearance, aroma, and taste attributes. Higher-grade materials corresponded to brighter liquor and smoother taste, whereas lower-grade materials exhibited darker liquor and increased astringency. These differences were used as the basis for subsequent analysis of chemical and microbial characteristics to explore how raw material grade contributes to quality differentiation in FBT.

### 3.2. Quality Components in Raw Dark Teas and Fu Brick Teas Across Grades

**Figure 2:**
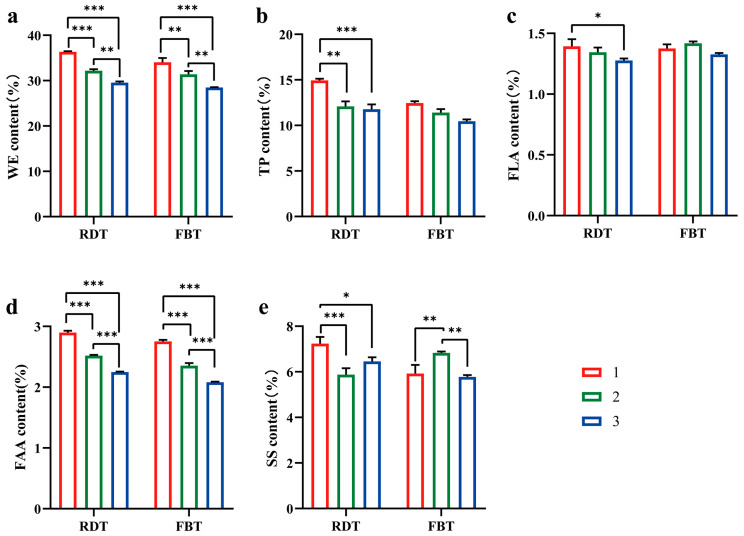
Quality-related components across grades in raw dark tea (RDT) and finished Fu brick tea (FBT). Panels depict WE (**a**), TP (**b**), FLA (**c**), FAA (**d**), and SS (**e**). Bars show means ± SD (*n* = 3 biological replicates per grade, unless stated otherwise). Group comparisons were evaluated using ANOVA; significance is denoted as * *p* < 0.05, ** *p* < 0.01, and *** *p* < 0.001. The details are provided in the Section 2.

Grade-dependent differences were observed in both the baseline composition of RDT and their transformation during processing into FBT (Figure 2). In the pre-fermentation raw material matrices (RDT), the content of WE (1M: 36.35 ± 0.14; 2M: 32.17 ± 0.36, 3M: 29.54 ± 0.27 (%)), TP (1M: 14.93 ± 0.19; 2M: 12.10 ± 0.5; 3M: 11.78 ± 0.53 (%)), and FAA (1M: 2.90 ± 0.03; 2M: 2.52 ± 0.01, 3M: 2.25 ± 0.01 (%)) decreased significantly with decreasing raw material grade (*p* < 0.05, 1M > 2M > 3M). These results indicate that higher-grade RDTs contained a greater proportion of extractable solids and nitrogenous compounds. The FLA content (1M: 1.39 ± 0.06; 2M: 1.34 ± 0.03, 3M: 1.27 ± 0.02 (%)) showed no significant difference among the three grades, suggesting relatively stable flavonoid accumulation before fermentation.

During the transition from RDT to FBT under standardized compression and flowering conditions, the concentrations of TPs and FAAs decreased across all grades, whereas SS increased, particularly in the lower-grade materials. FLA content remained relatively unchanged during this process. These compositional shifts reflect overall biochemical remodeling that occurs during post-fermentation. Among finished FBTs, C1M maintained significantly higher levels of WEs (C1M: 34.06 ± 0.92; C2M: 31.37 ± 0.76; C3M: 28.49 ± 0.06 (%)), TPs (C1M: 12.46 ± 0.20; C2M: 11.40 ± 0.39; C3M: 10.46 ± 0.10 (%)), and FAAs (C1M: 2.75 ± 0.02; C2M: 2.35 ± 0.04; C3M: 2.08 ± 0.10 (%)) than C2M and C3M (*p* < 0.05), while C2M exhibited the highest SS content, reflecting persistent grade-compositional differences after fermentation.

**Figure 3:**
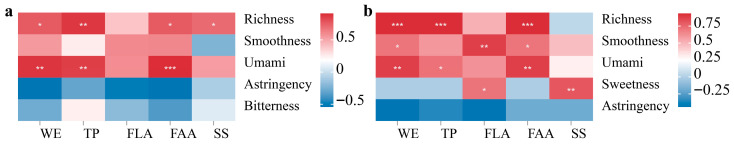
Composition–sensory Spearman correlations for (**a**) RDT and (**b**) FBT. Cells represent correlation coefficients between chemical indices (WE, TP, FLA, FAA, SS) and sensory attributes (richness/body, smoothness, umami/sweetness, astringency, bitterness). Computational settings are described in Section 2 (significance is denoted as * *p* < 0.05, ** *p* < 0.01, and *** *p* < 0.001).

Based on the observed compositional gradients among grades, Spearman correlation analyses were applied to assess the relationships between chemical components and sensory attributes in both RDTs and FBTs. Figure 3 presents Spearman correlations between chemical components and sensory attributes for RDTs (Figure 3a) and FBTs (Figure 3b). In both material grades, WEs, TPs, and FAAs were significantly and positively correlated with richness and smoothness, whereas astringency exhibited negative correlations with WEs and FAAs (*p* < 0.05). SS was weakly and positively associated with sweetness, while FLAs showed no consistent pattern across samples. The correlation strength increased after fermentation, particularly for TPs and FAAs in FBTs, indicating that compositional gradients were closely aligned with sensory differentiation. Notably, correlation coefficients increased following fermentation, particularly for TPs and FAAs in FBTs, indicating that chemical components became more closely aligned with sensory differentiation after processing.

Overall, the compositional differentiation among grades was consistent between raw materials and finished brick teas. Higher-grade samples showed higher content of extractable solids and phenolic compounds, whereas lower-grade samples contained more soluble sugars. These quantitative differences provide a chemical basis for subsequent analyses linking substrate composition with microbial community structure and sensory outcomes.

### 3.3. The Effect of Different Grades of Raw Materials on the Microbial Community Structure of FBT

#### 3.3.1. Fungal Community Structure Analysis

**Figure 4:**
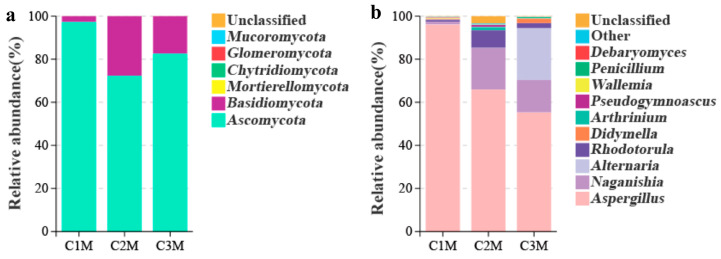
Fungal community composition of FBT by grade. The relative abundance of at (**a**) phylum and (**b**) genus level based on ITS amplicon sequencing; unassigned reads (<1%) were grouped as “other”.

To analyze the variations in the fungal communities and dominant taxa in FBTs produced from different raw material grade, ITS amplicon sequencing and culture medium enumeration were used [6,36]. A total of 1,128,318 valid sequences were generated form all samples, which were clustered into 6 phyla, 23 classes, 45 orders, 96 families, and 132 genera. At the phylum level, *Ascomycota* dominated across all samples (Figure 4a). At the genus level, *Aspergillus* was prevalent but showed a marked grade-dependent dominance gradient, accounting for 96.06% of the reads in C1M, 66.43% in C2M, and 55.01% in C3M (Figure 4b). As the contribution of *Aspergillus* declined, proportional increases were observed for *Naganishia* and *Alternaria*, along with several low-abundance genera. The culture medium enumeration supported these sequencing results. *A. cristatus* reached 2.73 × 10^6^ CFU g^−1^ in C1M, exceeding C2M (2.02 × 10^6^ CFU g^−1^) and C3M (1.69 × 10^6^ CFU g^−1^); all values were above the national lower limit (20 × 10^4^ CFU g^−1^, GB/T 9833.3-2013 [37]). The consistent patterns observed from both sequencing and cultivation confirmed *A. cristatus* as the dominant fungus in all grades of FBT, with relative abundance increasing with material grade.

Alpha diversity indices (ACE/Chao1 richness; Shannon and Simpson diversity) were used to assess evenness and richness within microbial communities. The pronounced genus-level dominance gradient implies corresponding differences in within-sample (α) diversity, with stronger dominance expected to reduce evenness. Consistent with this expectation, C1M exhibited lower Shannon (0.41 ± 0.03) and Simpson (0.08 ± 0.01) indices than C2M (1.67 ± 0.10; 0.52 ± 0.06) and C3M (1.83 ± 0.04; 0.62 ± 0.02) (Table 2; superscript letters denote *p* < 0.05), indicating strong dominance by a limited set of taxa. Richness estimates (ACE and Chao1) placed C1M and C3M at the higher end, with C2M being lower, suggesting that grade affects evenness more than the presence of low-abundance taxa. Because α-diversity is only within the brick structure, we next evaluated between-brick dissimilarity to determine whether grades also differ in the overall community configuration.

**Figure 5:**
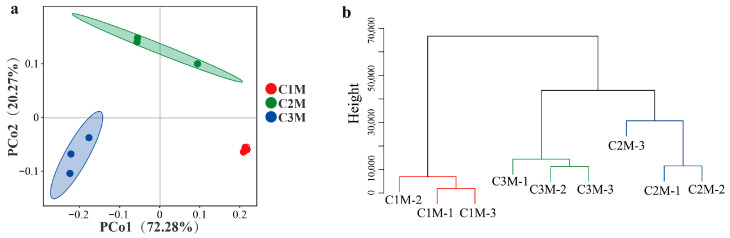
β-Diversity of fungal communities among FBT of different grades. (**a**) PCoA plot; (**b**) hierarchical clustering analysis (HCA) using Bray–Curtis distance.

Beyond within-sample metrics, between-sample (β) diversity was assessed to evaluate whether the grade also structured the overall community configuration among bricks. The β-diversity based on Bray–Curtis distances reflected the differences in fungal community composition and distribution between grades. PCoA showed compact grouping for C1M and greater dispersion for C2M/C3M (PCo1 = 72.28% and PCo2 = 20.27%; Figure 5a). C1M formed a compact assemblage with limited scatter around its centroid, whereas C2M/C3M exhibited broader dispersion, consistent with greater among-sample heterogeneity at lower grades. Hierarchical clustering based on the same distance metric produced three grade-level clusters, with C2M grouping closer to C3M than to C1M (Figure 5b). These ordinations indicate persistent grade-associated differences in the overall community configuration among the finished bricks.

The convergence of *Aspergillus* dominance (supported by CFU counts), α-diversity reductions aligned with stronger dominance, and β-diversity separation by grade identified a restricted set of taxa whose abundance tracked quality strata. Therefore, the subsequent analysis focused on differential and indicator taxa to determine which fungal groups most strongly accounted for the observed between-grade dissimilarity.

#### 3.3.2. Grade-Associated Differential Fungal Indicators Supported by Lineage Evidence and Pairwise Contrast

**Figure 6:**
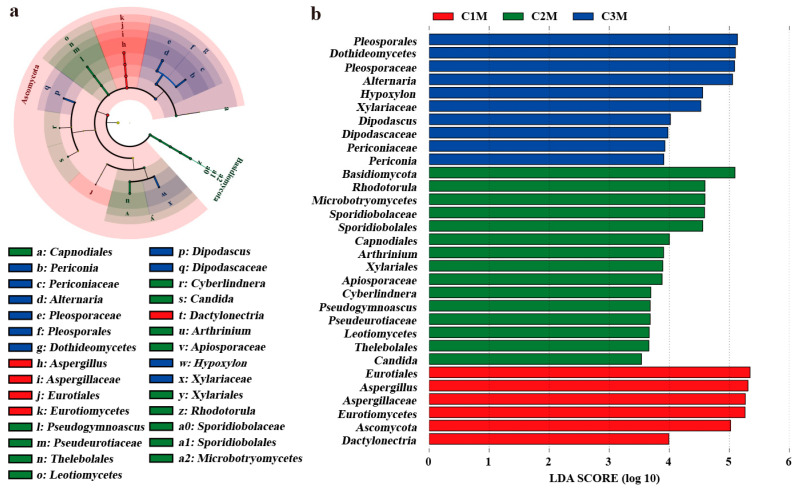
LEfSe analysis of differential fungal taxa among FBTs. (**a**) Cladogram of discriminant. (**b**) LDA score histogram for fungus. (Thresholds: LDA > 3.5, *p* < 0.05).

**Figure 7:**
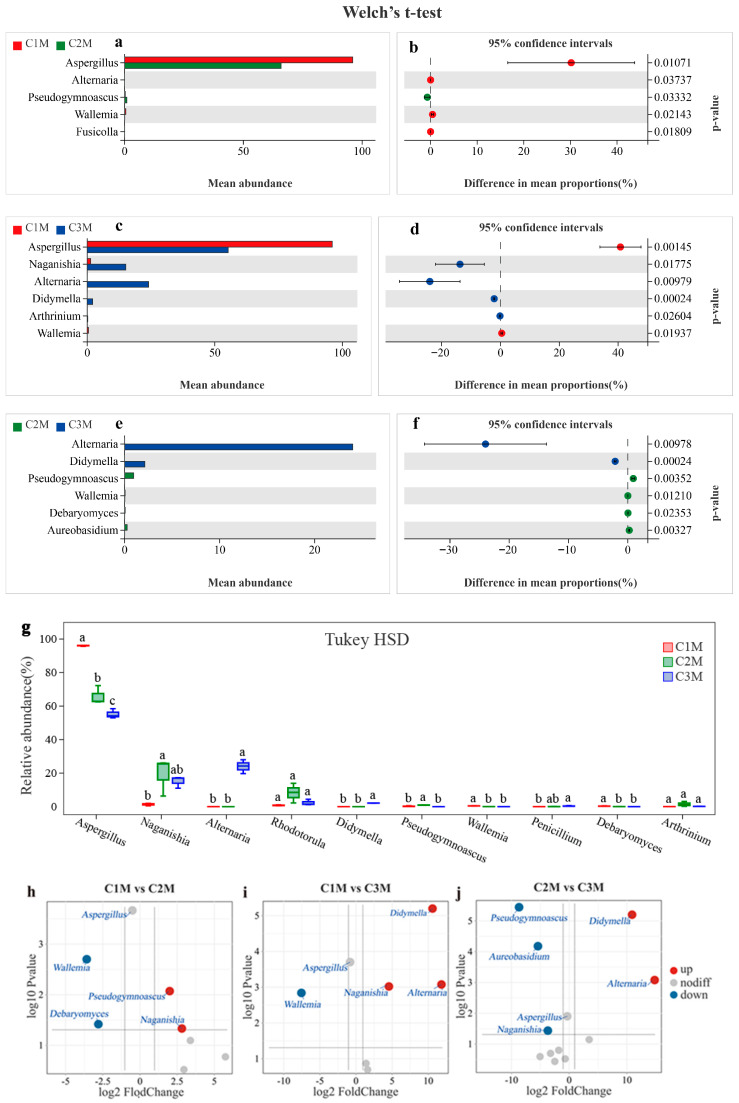
Pairwise contrasts of fungal genera across grades. (**a**–**f**) Welch’s *t*-test estimates of mean proportion differences with 95% CIs; (**g**) Tukey HSD test. Different superscript letters within a genus denote significant differences among grades (*p* < 0.05). (**h**–**j**) Volcano plots (log_2_ fold change vs. –log_10_ *p*) for C1M vs. C2M, C1M vs. C3M, and C2M vs. C3M. Volcano plots based on OPLS-DA. OTUs meeting the criterion of VIP > 1 were selected for comparative analysis.

Differential abundance analyses were performed to identify fungal taxa contributing to grade-dependent community differences (Figure 6 and Figure 7). Lineage-informed LEfSe analysis revealed distinct enrichment patterns across grades, with an LDA threshold of 3.5 and significance level of *p* < 0.05. LEfSe identified a coherent enrichment along the Aspergillus lineage in C1M, spanning *Ascomycota*, *Eurotiomycetes*, *Eurotiales*, *Aspergillaceae*, and the genus *Aspergillus* (Figure 6a,b). In contrast, C2M and C3M displayed a higher representation of yeasts and environmental molds, with prominent signals for *Naganishia*, *Rhodotorula*, *Alternaria*, *Pseudogymnoascus*, and clades within *Pleosporales* and *Xylariales*. The continuity of the *Aspergillus* signal across taxonomic ranks in C1M, together with diversified profiles in the lower-grade products, is consistent with the dominance gradient observed for community composition.

Pairwise comparisons further supported these lineage-based patterns. Across all three pairwise contrasts, the proportion of *Aspergillus* was significantly higher in C1M than in C2M and C3M, whereas that of *Naganishia* and *Alternaria* was proportionally higher in the lower-grade groups (Figure 7a–f). Volcano plots summarizing log_2_ fold change against −log_10_ *p* reproduced the same directions for C1M versus C2M, C1M versus C3M, and C2M versus C3M (Figure 7h–j). The concordance between lineage-guided and pairwise frameworks support *Aspergillus* as a robust indicator for the higher-grade product and *Naganishia*, *Alternaria*, and *Pseudogymnoascus* as indicators characteristic of the lower-grade products.

Based on these results, *Aspergillus* was defined as the primary indicator taxon for the higher-grade product, while *Naganishia*, *Alternaria*, and *Pseudogymnoascus* were selected as representative indicators for the lower-grade products. This indicator set was subsequently used as the focal panel for quantitative association analyses with quality-related constituents (WE, TP, FAA, and SS) and sensory attributes, enabling an integrated evaluation of microbiological signals in relation to flavor outcomes.

### 3.4. Correlation Between Key Fungal Taxa and Quality-Related Components of Raw Dark Tea and Fu Brick Tea

**Figure 8:**
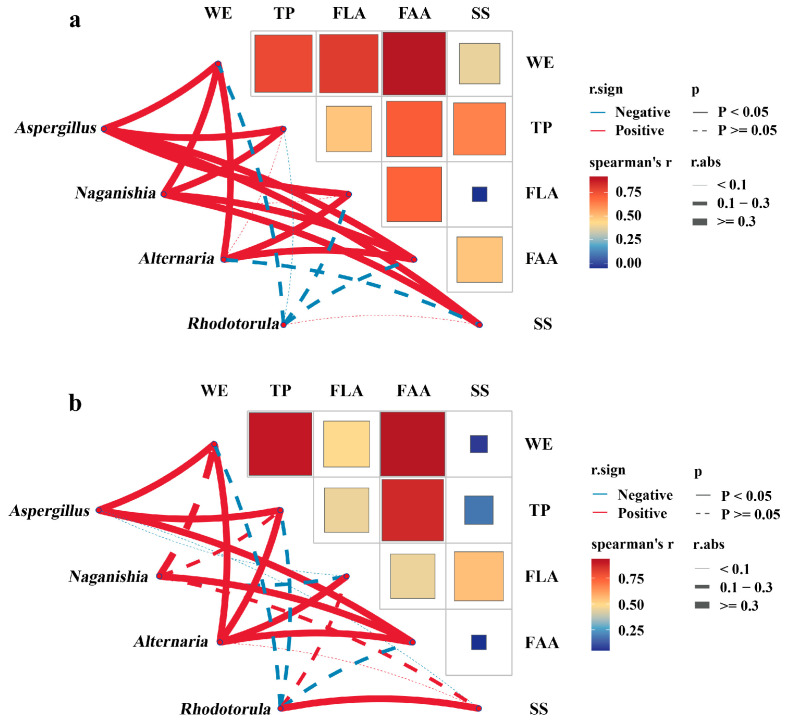
Two-layer correlation analysis linking raw-material composition, finished-brick microbiota, and product chemistry. (**a**) Spearman correlations between raw dark tea (RDT) constituents (WE, TP, FLA, FAA, SS) and the relative abundances of key genera quantified in the corresponding Fu brick tea (FBT). (**b**) Spearman correlations between these genera and chemical constituents of the same FBTs.

Based on the observed grade-dependent chemical differences in RDT and systematic shifts in the FBT microbiota, correlation analyses were conducted to examine association between the initial chemical composition of RDT and the relative abundance of major fungal taxa in finished FBTs, as well as the relationship between fungal community and the chemical profile of FBT. These sequential correlations were illustrated in Figure 8.

When RDT composition was correlated with the relative abundances of major fungal genera in FBTs (Figure 8a), significantly positive associations were observed between *Aspergillus* and RDT WE (|r| = 0.93, *p* < 0.05), TP (|r| = 0.88, *p* < 0.05), and FAA (|r| = 0.92, *p* < 0.05). By contrast, RDT SS showed weak or non-significant associated with *Rhodotorula* (|r| = 0.08, *p* > 0.05). *Naganishia* and *Alternaria* displayed moderate correlations with RDT WE and RDT FAA, whereas RDT FLA was weakly associated with all examined fungal genera. These data indicated that variation in the initial substrate composition covaried with the fungal community structure of the finished products.

When the same genera were correlated with the chemical components of FBT (Figure 8b), *Aspergillus* remains positively associated with FBT WEs (|r| = 0.78, *p* < 0.05), FBT TPs (|r| = 0.74, *p* < 0.05), and FBT FAAs (|r| = 0.93, *p* < 0.05), while its associations with FBT FLAs was weak. In contrast, *Rhodotorula* exhibited a positive association with FBT SS (|r| = 0.34, *p* < 0.05), whereas *Naganishia* and *Alternaria* showed weak but positive associations with FBT WEs and FAAs (coefficients in Figure 8b). These results indicate a consistent pattern in which the compositional characteristics of RDTs were reflected in the fungal community composition of FBTs ang resulting product chemistry.

Taken together, the direction and strength of correlations across Figure 8a,b support a sequential association linking substrate composition, fungal relative abundance, and product composition. Specifically, RDTs with higher WEs, TPs, and FAAs were associated with increased *Aspergillus* abundance in FBTs, which in turn corresponded to higher WE, TP, and FAA contents in the finished teas. By contrast, RDTs richer in SS were weakly associated with higher representation of *Rhodotorula* and elevated SS in the corresponding FBTs.

## 4. Discussion

The quality formation of FBT arises from complex biochemical and ecological processes driven by both substrate chemistry and microbial succession [6,10,11]. Although previous studies have extensively explored the microbial community during the flowering stage [15,17,18], the intrinsic variability of the RDT substrate and its role in steering fermentation outcomes remain less well understood. Recent multi-omics and chemometric studies on dark teas have highlighted that substrate composition plays a key role in defining microbial colonization and metabolite trajectories [16,19,23,31]. The present study reinforces this view by demonstrating that the substrate itself, through its compositional heterogeneity, is closely associated with microbial community configuration, which is reflected in product chemistry and sensory attributes. The enrichment of WEs, TPs, and FAAs in high-grade RDTs supported the development of teas with enhanced body, smoothness, and fungus aroma (Figure 3). Together, these results indicate that the flowering stage represents an ecologically structured succession in which substrate nutrient availability is aligned with microbial assembly and downstream quality expression, rather than a uniform colonization process [38,39,40,41].

The fungal community patterns observed across tea grades provide further evidence of substrate-mediated ecological filtering. Higher-grade FBTs were dominated by *Aspergillus cristatus*, whereas lower grades exhibited a greater representation of *Naganishia*, *Rhodotorula*, and *Alternaria* (Figure 4 and Figure 5, and Table 2). These patterns are consistent with earlier reports showing that polyphenol- and nitrogen-enriched matrices favor filamentous fungi capable of oxidative and proteolytic metabolism [8,13,21,22], while carbohydrate-enriched environments are more permissive for yeast-associated taxa [6,15,42]. The observed decline in TP and FAA content from RDT to FBT across grades likely reflects phenolic oxidation and peptide degradation [13,43], which is consistent with smoother taste and reduced astringency observed in higher-grade products (Figure 2 and Figure 3). Reduced α-diversity and compact β-diversity clustering in high-grade samples indicate an ecologically streamlined and more consistently configured consortium (Figure 4 and Figure 5), echoing findings from directed *Eurotium*/*Aspergillus cristatus*–dominated fermentations, in which functional dominance is associated with more stable sensory traits [44,45,46]. Similar community specialization has also been reported in Pu-erh and Qingzhuan teas, where controlled oxygen and moisture conditions promote adaptive microbial assemblages that optimize aroma and taste [27,28]. Together, these results support the concept that substrate composition acts as a primary determinant of microbial succession, analogous to substrate–microbe coupling described in other solid-state fermentations [40,47,48].

The sequential associations among RDT chemistry, fungal community composition, and FBT chemistry provide evidence for the propagation of substrate properties through fermentation stages. Positive correlations between WE, TP, FAA, and *Aspergillus* abundance in the finished bricks, together with the alignment between RDT composition and FBT microbiota and between microbiota and FBT chemistry (Figure 8), indicate that nutrient-richer substrates are associated with higher *A*. *cristatus* representation and higher WE/TP/FAA levels in the finished teas [17,25,49]. Conversely, SS-rich substrates were only weakly associated with *Rhodotorula* in the present dataset, and *Rhodotorula* showed a positive association with SS in FBTs, suggesting a sugar-linked ecological signal rather than a dominant driver of overall quality formation. Accordingly, the present results support a substrate-mediated association model in which the chemical environment of RDT is aligned with the microbial community structure during flowering, which is further aligned with product chemical profiles and sensory differentiation. This integrative view not only reconciles compositional and sensory hierarchies among FBT grades but also situates tea fermentation within a broader conceptual framework of nutrient-directed microbial assembly.

Nevertheless, it should be noted that the current study primarily infers these relationships from correlative evidence. Although compositional and microbial data consistently indicate substrate-driven differentiation, the specific enzymatic or genetic mechanisms underlying these processes remain unresolved. Future investigations combining enzymatic assays, time-resolved ITS amplicon sequencing, meta-transcriptomics, and metabolite tracing will be essential to clarify whether these compositional shifts are mediated by specific fungal enzyme systems or transcriptional regulators [50,51,52]. In addition, time-resolved sampling across the flowering stage, coupled with targeted metabolomics and functional omics, would allow direct testing of whether the observed “RDT—microbiota—chemistry” alignment reflects causal dependencies under production-relevant conditions in the field. Expanding this framework to include time-resolved multi-omics datasets may ultimately enable predictive control of fermentation pathways and rational grading of raw materials in dark tea manufacturing.

## 5. Conclusions

This study demonstrates a substrate-mediated association framework underlying quality differentiation in FBT. Variations in the chemical composition of RDT were mirrored in fungal community structure and quality-related metabolites. High levels of WEs, TPs, and FAAs favored the dominance of *Aspergillus cristatus*, producing teas with greater body, smoothness, and fungus aroma, while sugar-rich substrates supported increased representation of *Rhodotorula* and *Naganishia*, leading to sweeter but less complex profiles. These findings reveal that the substrate acts as an ecological filter guiding microbial succession and metabolic transformation, providing a compositional basis for quality differentiation and process guidance rather than deterministic causality. Future studies integrating enzyme-level assays with time-resolved multi-omics approaches will further clarify how substrate composition, fungal activity, and sensory outcomes are aligned, advancing a more refined understanding of fermentation regulation in dark tea production.

## Figures and Tables

**Table 1:** Sensory evaluation of raw dark tea (RDT, 1M–3M) and paired Fu brick (FBT, C1M–C3M).

Sample	Appearance	Liquor Color	Amora	Taste	Leaf Base
1M	Tight and heavy, black auburn	Orange	Pure and normal	Mellow with thick	Yellowish auburn
2M	Tight, yellowish auburn	Orange	Pure and normal	Mellow	Yellowish auburn
3M	Tight, brownish auburn	Light orange	Less pure	Mellow with astringent	Brownish auburn
C1M	Flat brick surface, with golden florals blooming in profusion	Orange red bright	Pure and normal, more strong fungus aroma	Heavy and more mellow, brisk and smooth, have fungus taste	Brown auburn
C2M	Flat brick surface, with golden florals blooming in profusion	Orange red bright	Pure and normal, strong and lasting fungus aroma	Mellow and thick, fresh and smooth, with fungus taste	Black auburn
C3M	Flat brick surface, with golden florals blooming in profusion	Orange red moderately bright	High	Heavy and mellow	Black auburn

Appearance, liquor color, aroma, taste, and leaf base were assessed following GB/T 23776-2018.

**Table 2:** α-Diversity indices of fungal communities in FBT produced from graded raw materials.

Sample	ACE	Chao1	Shannon	Simpson
C1M	189.66 ± 21.96	182.06 ± 30.69 ^a^	0.41 ± 0.03 ^c^	0.08 ± 0.01 ^c^
C2M	143.48 ± 30.57	120.69 ± 16.65 ^b^	1.67 ± 0.10 ^b^	0.52 ± 0.06 ^b^
C3M	184.42 ± 17.81	179.07 ± 12.27 ^ab^	1.83 ± 0.04 ^a^	0.62 ± 0.02 ^a^

ACE, Chao1, Shannon, and Simpson (mean ± SD, *n* = 3). Different superscript letters within a column denote significant differences among grades (*p* < 0.05).

## Data Availability

The original contributions presented in this study are included in the article. Further inquiries can be directed to the corresponding authors.
